# pH-Controlled Release of Antigens Using Mesoporous Silica Nanoparticles Delivery System for Developing a Fish Oral Vaccine

**DOI:** 10.3389/fimmu.2021.644396

**Published:** 2021-04-19

**Authors:** Weibin Zhang, Chunhua Zhu, Fangnan Xiao, Xiaodong Liu, Anhua Xie, Fangman Chen, Panpan Dong, Pingdong Lin, Chenyang Zheng, Hong Zhang, Hui Gong, Yunkun Wu

**Affiliations:** ^1^ Provincial University Key Laboratory of Cellular Stress Response and Metabolic Regulation, College of Life Science, Fujian Normal University, Fuzhou, China; ^2^ Institute of Animal Husbandry and Veterinary Medicine, Institute of Biotechnology, Fujian Academy of Agricultural Sciences, Fuzhou, China; ^3^ State Key Laboratory of Structural Chemistry, Fujian Institute of Research on the Structure of Matter, Chinese Academy of Sciences, Fuzhou, China

**Keywords:** vaccine, nanoparticle, oral delivery, large yellow croaker (*Larimichthys crocea*), immune response

## Abstract

The development of effective vaccines and delivery systems in aquaculture is a long-term challenge for controlling emerging and reemerging infections. Cost-efficient and advanced nanoparticle vaccines are of tremendous applicability in prevention of infectious diseases of fish. In this study, dihydrolipoamide dehydrogenase (DLDH) antigens of *Vibrio alginolyticus* were loaded into mesoporous silica nanoparticles (MSN) to compose the vaccine delivery system. Hydroxypropyl methylcellulose phthalate (HP55) was coated to provide protection of immunogen. The morphology, loading capacity, acid-base triggered release were characterized and the toxicity of nanoparticle vaccine was determined *in vitro*. Further, the vaccine immune effects were evaluated in large yellow croaker *via* oral administration. *In vitro* studies confirmed that the antigen could be stable in enzymes-rich artificial gastric fluid and released under artificial intestinal fluid environment. *In vitro* cytotoxicity assessment demonstrated the vaccines within 120 μg/ml have good biocompatibility for large yellow croaker kidney cells. Our data confirmed that the nanoparticle vaccine *in vivo* could elicit innate and adaptive immune response, and provide good protection against *Vibrio alginolyticus* challenge. The MSN delivery system prepared may be a potential candidate carrier for fish vaccine *via* oral administration feeding. Further, we provide theoretical basis for developing convenient, high-performance, and cost-efficient vaccine against infectious diseases in aquaculture.

## Introduction

Aquaculture has grown rapidly in the 21st century due to human population growth and declining marine capture in wild fisheries. According to Food and Agriculture Organization statistics, global aquaculture production is close to 100 million tons (1). Large yellow croaker (*Larimichthys crocea*) is the largest mariculture fish in China and in 2017, the annual production of large yellow croaker in marine fish has reached 177,640 tons (2). Due to long-term irregular farming, inbreeding, and nutritional imbalances, the risk of emerging and reemerging disease outbreaks in large yellow croaker has increased. The most prevalent diseases affecting large yellow croaker are white-gill disease, vibriosis, iridovirus disease, etc., with diseases caused by bacteria being the most serious due to their high incidence and wide epidemic proportions (3–5). This has threatened and challenged the aquaculture industry and food safety. It has been reported that the diseases caused by *Vibrio parahaemolyticus*, *Vibrio alginolyticus*, and *Vibrio harveyi* have become the major constraint to the sustainable development of large yellow croaker aquaculture (3, 6, 7). The traditional treatment of bacterial diseases is mainly through antibiotics. However, the use of antibiotics will not only produce drug residues in fish, but also endanger the health of consumers. Moreover the large-scale use of antibiotics cannot treat and prevent all aquatic diseases well, and may even pollute the water. Vaccination is the most effective approach of preventing infectious diseases in fish. The use of vaccines can reduce the risk of infection from viruses, bacteria, or parasites, reducing economic losses while ensuring the healthy development of fish farming and maintaining food safety. Therefore, fish vaccine research and development is considered to be the most promising way to solve various types of aquatic diseases, reducing drug residues, and improve the quality of aquatic products. There is currently an urgent need to develop a more convenient, safe, and efficient vaccine and delivery system for controlling emerging and reemerging infectious diseases (8, 9). The immune mechanisms among different fish species are diverse, bringing greater difficulties to fish vaccine design, but also opportunities. Therefore, the design of high-performance, cost-effective, and stable vaccines with better release kinetics have tremendous application prospects.

Vaccine development has evolved from traditional whole-pathogen vaccines to the use of only single proteins and peptides as antigens. The new problem is that these antigens have a greatly reduced immunogenicity when used alone and do not achieve the desired level of immune protection (10). It is necessary to develop adjuvants and effective delivery systems to further enhance the immunogenicity of antigens and their application to actual production (11). An adjuvant is an immune agent that activates antigen-presenting cells and triggers a strong immune response (12), while causing less toxicity and side effects to the body itself and providing long-term protection. The advantage of carrier systems is that they minimize antigen degradation by encapsulation, achieve controlled antigen release, enhance bioavailability, and transmit the antigen to target immune cells while protecting it (13, 14). In recent years, the effectiveness of nanoparticle vaccination has been widely verified by using nanoparticles as adjuvant and carrier systems to protect antigens of aquatic vaccines (10, 15). Nanoparticles have a suitable size, so they can be absorbed by endocytosis, promoting cell antigen absorption and enhancing antigen presentation ability (15, 16). At present, nanoparticles used in the development of aquatic vaccines mainly include nanoliposomes (15), macromolecule nanoparticles (17), inorganic nanoparticles (18), and immunostimulatory complexes (ISCOMs) (19). Due to their beneficial physical and chemical properties, inorganic nanoparticles have been used in vaccine research and development as adjuvants and potential vaccine carrier systems (12, 20). Mesoporous silica nanoparticles (MSN) are inorganic and an ideal carrier for vaccine delivery, which is tolerated well in animals (18, 21). Studies have confirmed that MSN delivery system was high performing, with a high loading degree, controllable release, and a good immune adjuvant effect. Additionally, MSN materials could elicit humoral and cell-mediated immune responses, and consequently MSN plays an important role in the design of novel vaccine delivery systems (12, 18, 22).

We previously reported an oral vaccine with an intelligent carrier, poly [(methyl methacrylate)-co-(methyl acrylate)-co-(methacrylic acid)]-poly (d, l-lactide-co-glycolide) (PMMMA-PLGA) that was capable of effectively targeting surface immunogenic protein (SIP) to the rear intestine, eliciting a robust immunity in tilapia (17). However, commercial PLGA, the carrier ingredient of the vaccine is expensive, which limits its application in large-scale farms. Fish breeding is relatively low-cost, so it is crucial to develop cost-effective nanocarriers to reduce the cost of vaccine development and industrialization. Dihydrolipoamide dehydrogenase (DLDH) is a cross protective immunogen of *Vibrio harveyi*, *Vibrio alginolyticus*, and *Vibrio parahaemolyticus* in large yellow croaker (4). It is a model antigen for economical fish production and conveys good immune protection against the *Vibrio* species. In this study, the DLDH protein of *Vibrio alginolyticus* was expressed by genetic engineering, and the purified antigen was loaded into mesoporous silica materials and assembled into nanoparticles. Hydroxypropyl methylcellulose phthalate (HP55) served as coatings for the nanoparticle, providing protection for MSN-DLDH nanoparticles. After assembly, the properties of the nanoparticle vaccine were further characterized and evaluated by *in vitro* and *in vivo* experiments. *In vitro* studies confirmed that the nanoparticles were pH-controlled release of antigens and non-toxic to the kidney cells of large yellow croaker. Further, the vaccine was administered orally by feeding to large yellow croaker. Our study demonstrated that the cytokines of IFNγ, IL-1β, IL-2, IL-4, and IL-13 in difference immune tissues (intestine, spleen, and head kidney) were significantly up-regulated at different time *via* oral administration. And the levels of serum antibody against large yellow croaker steadily increased. Our study suggested that the vaccine is capable of eliciting both innate and adaptive immune response and providing protection for large yellow croaker, with the relative percent survival (RPS) of 76.92%. This study provides a theoretical basis for the future development and clinical application of economical oral vaccines based on the MSN delivery system.

## Materials and Methods

### Materials

The experimental large yellow croaker (about 20 g, randomly selected) was purchased from Ningde Fufa Fisheries Co., Ltd. (Ningde city, Fujian province, China). The kidney cell of large yellow croaker (YCK), *Vibrio alginolyticus* and the monoclonal antibody 2H_5_F_4_ against large yellow croaker were donated by professor Gong Hui from Institute of Biotechnology, Fujian Academy of Agricultural Sciences. HP55 and span 80 were purchased from Sinopharm Chemical Reagent Co., Ltd. (Shanghai, China). N, N, N-trimethylhexadecan-1-aminium 4-methylbenzenesulfonate (CTATos), horseradish peroxidase (HRP) conjugated goat anti-mouse IgG, and o-phenylenediamine (OPD) were purchased from Sigma-Aldrich (St. Louis, USA). Triethanolamine (TEAH_3_), tetraethyl orthosilicate (TEOS), polyvinyl alcohol (PVA) and triethyl citrate were purchased from Aladdin (Shanghai, China). CellTiter-Lumi™ plus luminescent cell viability assay kit was purchased from Beyotime (Shanghai, China). TRIzol™ Reagent was purchased from Thermo Fisher Scientific (Beijing, China). Primescript™ RT Reagent kit with gDNA Eraser (RR047A) and TB Green™ Premix Ex Taq™ II kit (RR820A) were purchased from TaKaRa (Dalian, China).

### Gene Cloning and Protein Expression

The *DLDH* gene (GenBank: MK281384) was amplified by PCR with a hexa-histidine TEV protease digestion site added to the 5′ end and then subcloned into the pET-28a vector. The recombinant plasmid was transformed into *E. coli BL21 (DE3)* for expression. After culturing for 3 h in LB, IPTG (BBI Life Science) was added to the media at a final concentration of 0.3 mM for induction of the target protein at 16°C for 12 h. The cells were collected by centrifugation at 6,720 *g* for 10 min, cells were broken by an ultrasonic cell disruptor in buffer containing 50 mM Tris pH 8.0, 300 mM NaCl, 0.1% Tween-20 and 5% glycerol. Cell fragments were removed by centrifugation at 98,900 *g*, and supernatant was purified by nickel-nitrilotriacetic acid (Ni^2+^-NTA) chromatography column (GE Healthcare). The His tag was removed by TEV protease digestion and dialysis at 4°C overnight, and the overexpressed DLDH proteins were harvested by Ni^2+^-NTA column for the second time. The purified protein was analyzed by sodium dodecyl sulphate-polyacrylamide gel electrophoresis (SDS-PAGE). Total DLDH proteins were concentrated to approximately 5 mg/ml with a 30 kDa cut off ultracentrifugal filter device (Amicon, Millipore).

Swiss-model on line server was used for homology modeling to build the three-dimensional structure of DLDH. The DLDH protein from *Colwellia psychrerythraea* 34H (PDB ID 3IC9, 60.17% identity) was used as a template for modeling, and the three-dimensional structure diagram was performed using PyMOL software (23, 24).

### Preparation of Nanoparticles

**Figure 1:**
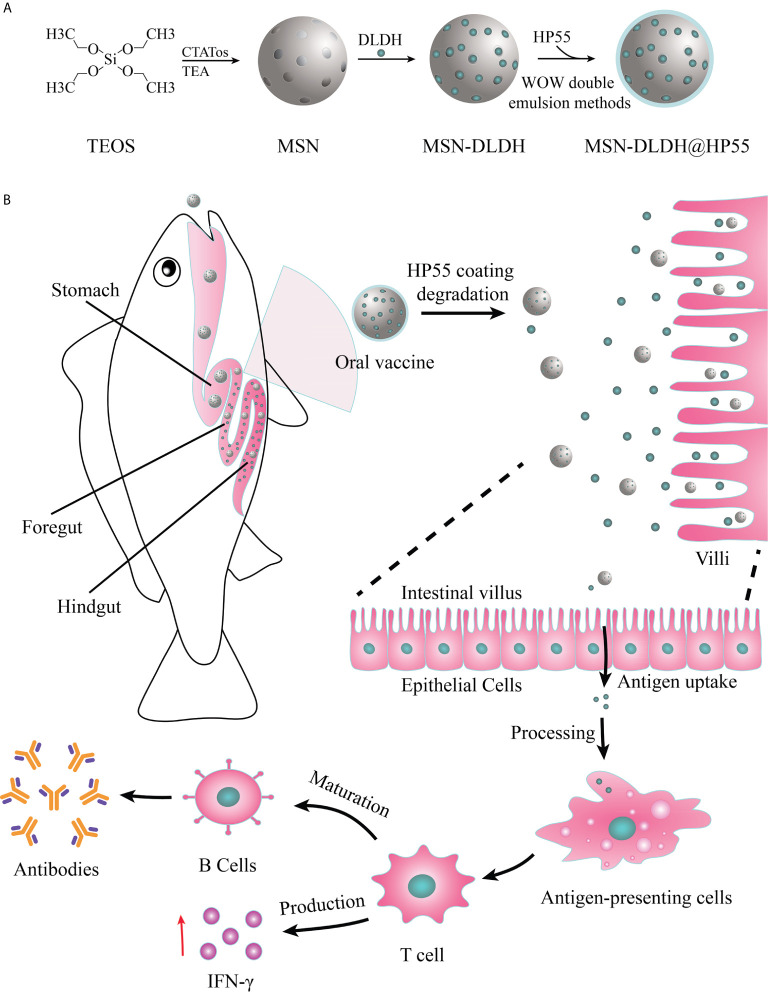
Schematic of preparation and oral administration of nanovaccines. **(A)** Schematic representation of the preparation process of MSN-DLDH@HP55 nanovaccines. **(B)** Schematic of the delivery of DLDH antigens using a mesoporous silica nanoparticle delivery system *in vivo* and evoking immune response.

MSN preparation was performed according to previously described protocols with minor modification (25). CTATos (4.80 g), 0.70 ml TEAH_3_, and 250 ml water were mixed in a round bottom flask and stirred at 80°C for 1 h to dissolve. Then, 10 ml TEOS was added quickly, and stirring was continued for 2 h. MSN was obtained by extracting the product three times with 6 g/L NH_4_NO_3_ methanol solution at 60°C. Then MSN and DLDH were mixed in the buffer (50 mM Tris, pH 8.0, 300 mM NaCl, 0.1% Tween-20, 5% glycerol) and at the ratio of 1:1.28 (m_MSN_/m_DLDH_), and then gently rotated at 7°C overnight, and the protein was loaded into the MSN, resulting in MSN-DLDH. The loading degree of the protein in MSN-DLDH was calculated by the following formula: DLDH load ratio (%) = DLDH_load/_(DLDH_load_ + MSN_mass_) × 100%. In order to protect the antigen from destruction by strong acid environment in the stomach, HP55 enteric coatings were prepared by the double emulsion method (26). The MSN-DLDH@HP55 vaccine was obtained after two phacoemulsification and deposition steps (**Figure 1A**). Briefly, internal aqueous phase (W1) MSN-DLDH@HP55 suspension, protective agent PEG400 and emulsifier Span 80 were mixed with oil phase (O, CH_3_COOCH_2_CH_3_/CH_2_Cl_2_, v/v=1:1). The primary emulsion (W1/O) was obtained after ultrasonic emulsification at 475 W for 2 min. The primary emulsion was transferred to the external aqueous phase W2 (containing 0.1% PVA), and the complex emulsion (W1/O/W2) was obtained after ultrasonic emulsification at 160 W for 3 min. Then, the mixture of CH_2_CH_2_OH and CH_3_COCH_3_ (v/v= 1:1, containing 0.5% HP55) was added slowly under stirring. MSN-DLDH@HP55 vaccine was obtained by centrifugation at 17300 g for 10 min after stirring at room temperature until the organic solvent evaporated. Subsequently, the vaccine was attached to the feed, adhering to detailed method as follows: The commercial feed of large yellow croaker was mixed with the binder, and then the vaccine suspension was sprayed on the feed evenly. Finally, the feed was soaked in the chitosan solution (0.5% chitosan, 2.5% triethyl citrate, 5% acetic acid) for 3 s to form coatings and quickly transferred to distilled water for 5 s, and then dried using vacuum drying technique.

### Characterization Analysis of Materials

The morphology of sample was analyzed by field emission transmission electron microscope (FEI Tecnai F20, USA) at an operating voltage of 200 kV. The sample was dispersed evenly in ddH_2_O, and the droplets of sample suspension were air-dried on the copper grid. The CHNS elemental analysis was performed using an Elemental Analyzer (Vario EL-Cube, Elementar, GER). The particle potential of the sample was analyzed by laser particle size and zeta potential analyzer (BI-200SM, Brookhaven, USA). The samples were dissolved in ddH_2_O (1 mg/ml) and measured immediately at 25°C for five times, 3 s/round. The loading capacity of the material was analyzed by thermal gravimetric analyzer (DSC, Netzsch, STA 449C, Germany). The sample was heated in nitrogen atmosphere, ranging from 35°C to 900°C, at 10°C/min heating rate. The specific surface area and porosity of the sample were analyzed by chemisorption-mass spectrometry (Hiden, ASAP2020C+M, USA). The specific surface area was calculated by the Brunauer-Emmett-Teller (BET) equation; the pore size distribution was calculated using the Barrett-Joyner-Halenda (BJH) model; and the pore volume was determined by the amount of adsorption at relative pressure (P/P0) of 0.97.

### Acid-Base Release Characteristics

The acid-base release performance of MSN-DLDH@HP55 was analyzed by artificial gastric fluid at pH 1.2 and artificial intestinal fluid at pH 7.5. The MSN-DLDH@HP55 vaccine suspension was centrifuged, and the supernatant was discarded. Then, the pellet was resuspended in artificial gastric and intestinal fluid, respectively. Both groups were incubated in a shaker at 30°C for 6 h at 260 rpm. The control MSN-DLDH was incubated in artificial intestinal fluid under the same condition. In order to determine whether the vaccine could release the antigen normally after treatment in an acidic environment, the vaccine was incubated in artificial gastric fluid at pH 1.2 for 3 h and then transferred to artificial intestinal fluid at pH 7.5 for 3 h. The DLDH concentration in the supernatant was measured by ultramicro ultraviolet visible spectrophotometer (Biochrom, NanoVue Plus, USA) every 30 min for each group.

### *In Vitro* Cell Cytotoxicity Assay

The cytotoxicity of DLDH proteins and MSN-DLDH@HP55 *in vitro* were assessed by CellTiter-Lumi™ plus luminescent cell viability assay kit (Shanghai, China). YCK cells were grown in 96-well plates (10^4^/well) in M199 media supplemented with 10% fetal bovine serum for 24 h at 27°C. Then YCK cells were incubated with different concentrations (0, 4, 8, 10, 20, 40, 60, 80, 100, 120, 140, 160, 180, 200 µg/ml) of DLDH proteins and MSN-DLDH@HP55 for 24 h, respectively. To each well, 100 μl CellTiter-Lumi™ plus solution was added and wells were shaken at room temperature for 2 min to lyse the cells. Next the plates were incubated at 25°C for 10 min. The luminescence of each well was measured by microplate reader (Biotek, Synergy), and cell viability was evaluated according to the following equation: Cell viability (%) = (Sample/Control) × 100%.

### Relative Percent Survival Assay

The RPS assay was performed to demonstrate the immune protection effects against *Vibrio alginolyticus*. The healthy fish were randomly divided into four groups (50 fish per group), each group had three biological repeats. The nanovaccine group and DLDH group were orally administered the MSN-DLDH@HP55 vaccine feed and DLDH vaccine feed, respectively, two times at 1 week interval, while the challenge group was given commercial feed all the time. Each time, the nanovaccine group was given 2 μg of the vaccine per gram of large yellow croaker each time of immunization. One week after the second stimulation, the challenge group, nanovaccine group and DLDH group were challenged with 10-fold median lethal dose (1.1 × 10^7^ CFU/ml) of *Vibrio alginolyticus* in large yellow croaker, while the immunized fish was intraperitoneal inoculated with 0.2 ml PBS as control. The relative percentage survival (RPS) of the large yellow croaker was recorded for consecutive 14 days and the RPS was calculated according to the following formula (27):

RPS=[1-(mortality in immunized group/mortality in control group)]×100%

### Analysis of Serum Antibody Levels

Three fish were randomly selected from each group for serum collection at day 0, 7, 14, and 21. Immune response was assessed by antibody serum levels determined by blocking ELISA (28). A 96-well was coated with 20 μg/ml of purified DLDH protein. The primary antibody was antiserum of large yellow croaker (1:50), the secondary antibody was monoclonal antibody 2H_5_F_4_ against large yellow croaker (1:500), and the third antibody was goat anti-mouse IgG conjugated to HRP (1:5,000). The optical density (OD) value of each well was determined by microplate spectrophotometer (BioRad, xMark). The OD value was transformed to percent of inhibition (PI) of the antibody in serum, determined with the following equation: PI (%) = (1-OD_490_ of sample serum/OD_490_ of control serum) ×100%. When the absolute value of the PI value was no less than 18.4%, the sample was determined to be positive. All tests were performed in triplicate.

### Analysis of the Expression Levels of Cytokines

Samples of intestine, spleen, and head kidney were collected on days 7, 14, 21 and 28. The total RNA of the sample was extracted by the Trizol reagent (29), and then the cDNA of the sample was prepared by reverse transcription using PrimeScript™ RT reagent kit with gDNA Eraser (RR047A). The relative expression levels of IFNγ, IL-1β, IL-2, IL-4, and IL-13 genes in samples were determined *via* reverse transcription-quantitative polymerase chain reaction (RT-qPCR) using TB GreenTM Premix Ex Taq™ II kit (RR820A). β-actin was used as an internal control with β-actin forward and reverse primers. The relative gene expression of each group was compared with that of day 0 according to the formula: Relative expression = 2^−ΔΔCt^ (30), where ΔCt = sample Ct value − β-actin Ct value, and ΔΔCt = immunized group ΔCt − control group ΔCt. Significance was determined by the t test (GraphPad Prism 8, unpaired t test). All data were tested in triplicate. The primers used for the RT-qPCR assay are listed in **Table 1**.

**Table 1:** The primers used for RT-qPCR assay.

Gene	Primer	Sequence (5’ to 3’)
β-actin	Forward	GACCTGACAGACTACCTCATG
	Reverse	AGTTGAAGGTGGTCTCGTGGA
IFN-γ	Forward	GTGATGATGATGATGATGATG
	Reverse	GCAGAAGAACCTGAATGTA
IL-1β	Forward	GGCTGAACCTTAGTACCCTTG
	Reverse	GATGTTGAAGTTTCTGTGGCG
IL-2	Forward	CTGCTGTGAGAAGGAACT
	Reverse	GCCAGGTGGATGAATGTA
IL-4	Forward	TCATCAGAACCAGACCAG
	Reverse	TTATCCGCACATTCAGAGA
IL-13	Forward	CGTCGATGGCAGAAATATTAACTG
	Reverse	GAGTACGGGTATTGGTCTTTCC

## Results

### Gene Clone and Protein Expression

**Figure 2:**
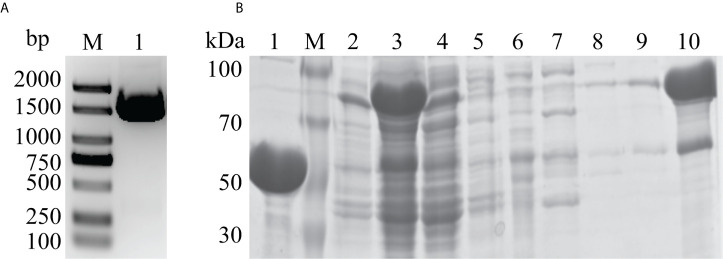
Gene cloning and protein purification. **(A)** PCR amplification of the *DLDH* gene. Lane M: 2 KB DNA marker; Lane 1: 1500 bp *DLDH* gene product. **(B)** SDS-PAGE analysis of DLDH purification by Ni^2+^-NTA column. Lane 1: After removal of His-tag by TEV protease and elution with 20 mM imidazole; Lane M: Protein marker; Lane 2: Precipitation; Lane 3: Supernatant; Lane 4: Flowthrough; Lanes 5–9: Fractions eluted with 0, 10, 20, 30, and 40 mM imidazole, respectively; Lane 10: Target protein eluted with 300 mM imidazole.

The *DLDH* gene was amplified by PCR, and the size was determined to be about 1500 bp (**Figure 2A**). The results of purified DLDH protein were shown in **Figure 2B**. Fractions were gradient eluted with imidazole using Ni^2+^-NTA column, and the DLDH protein was eluted with 300 mM imidazole. The His tag was removed by the TEV protease. A band of overexpressed DLDH protein, about 50 kDa in size, was separated and observed by the SDS-PAGE gel sample.

### Characterization of Analysis of Materials

**Figure 3:**
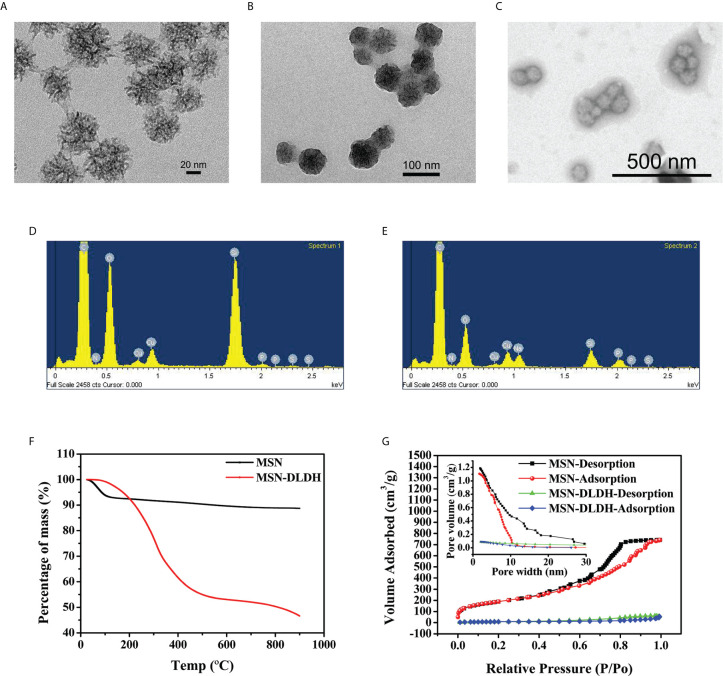
Characterization of the nanoparticles for DLDH protein delivery. TEM micrograph images: **(A)** MSN, **(B)** MSN-DLDH, and **(C)** MSN-DLDH@HP55; Energy dispersive X-ray spectroscopy analysis: **(D)** MSN and **(E)** MSN-DLDH; **(F)** Analysis of protein loading rate by thermo gravimetric analysis; **(G)** The nitrogen adsorption-desorption isotherms and pore size distributions of the MSN and MSN-DLDH.

The properties of nanoparticles were characterized by transmission electron microscopy (TEM), energy dispersive X-ray spectroscopy (EDX), dynamic light scattering, thermo gravimetric analysis (TGA) and nitrogen adsorption-desorption analysis. The results of TEM showed that MSN had a circular divergent pore structure with a diameter size of 71.39 ± 8.00 nm (**Figure 3A**). After the MSN was loaded with DLDH protein, the pore structure disappeared, and the pore was occupied by protein, with the size of 72.49 ± 8.07 nm (**Figure 3B**), which was consistent with the hydrodynamic particle size (81.6 nm) measured by dynamic light scattering (**Figure S2B**). The morphology of MSN-DLDH coated with HP55 was an irregular sphere, and each particle contained one or more MSN-DLDH units (**Figure 3C**). The larger particle size of MSN-DLDH indicated that DLDH adhered to the surface of MSN, and thus, enlarged the particle size. Moreover, EDX spectra analysis showed that the element of Si was decreased, and the O and P elements were increased after loading DLDH antigen (**Figures 3D, E**). CHNS analysis also confirmed that the contents of C and H elements in the MSN increased after loading, suggesting that DLDH was successfully loaded into MSN (**Table S1**). The zeta potentials of MSN and MSN-DLDH measured were −6.62 ± 4.27 mV and −20.27 ± 1.47 mV, respectively (**Figures S2C** and **D**). The load capacity of MSN was analyzed by TGA (**Figure 3F**). The mass losses of MSN and MSN-DLDH throughout the TGA process were 11.26% and 53.41%, respectively. Thus, the loading degree of DLDH protein in MSN was 42.15%, which was close to the theoretical value (43.32%) calculated by the previous formula. The specific surface area and porosity of MSN and MSN-DLDH were analyzed by nitrogen adsorption-desorption isotherms (**Figure 3G**). The specific surface area of MSN was 679.90 m^2^/g, the pore volume was 1.13 cm^3^/g, and the pore diameter was 7.83 nm. The specific surface area of MSN-DLDH was 29.38 m^2^/g, the pore volume was 0.06 cm^3^/g, and the pore diameter was 6.67 nm. The specific surface area, pore volume, and pore diameter of MSN decreased with the combination of protein, with the specific surface area decreased of 650.52 m^2^/g, the pore volume decreased of 1.07 cm^3^/g, and pore diameter decreased of 1.16 nm.

### Acid-Base Release Characteristics

**Figure 4:**
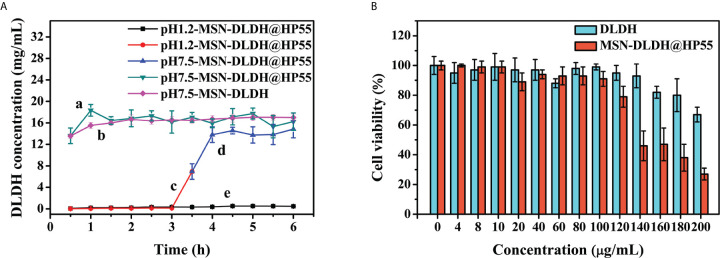
*In vitro* assay of nanoparticles. **(A)** Acid-base triggered release of MSN-DLDH@HP55. The results are presented as mean values (n=3) and error bars represent ± SEM. **(B)** Analysis of cytotoxicity of DLDH and MSN-DLDH@HP55 *in vitro* by CellTiter-Lumi™ plus luminescent cell viability assay kit. Cell viability of YCK cells was assessed after incubation with different concentrations (0, 4, 8, 10, 20, 40, 60, 80, 120, 140, 160, 180 and 200 µg/ml) of DLDH and MSN-DLDH@HP55 for 24 h, respectively. The results of cell viability are presented as mean values (n=3) and error bars represent ± SEM.

Analysis of acid-base release performance of the vaccine is shown in **Figure 4A**. In order to confirm whether the vaccine could release antigen after treatment in acidic environments, MSN-DLDH@HP55 vaccine was placed in artificial gastric fluid, and then in artificial intestinal fluid. MSN-DLDH@HP55 vaccine was incubated in artificial gastric fluid at pH 1.2 for 6 h, and the concentration of released DLDH was no more than 1 mg/ml. MSN-DLDH@HP55 was incubated in artificial intestinal fluid at pH 7.5 for 1 h, the release of DLDH was maximum, leading to a concentration that was higher than 13 mg/ml. The results showed that during the incubation in artificial gastric fluid at pH 1.2, the enteric coatings of HP55 did not dissolve, and the DLDH protein was not significantly released. However, during incubation in artificial intestinal fluid at pH 7.5, the enteric coatings gradually dissolved, and the DLDH protein was slowly released. Our results confirm that the enteric-coated MSN-DLDH@HP55 nanoparticles could be stable in acidic conditions and released the loaded DLDH protein in weak alkaline conditions.

### *In Vitro* Cytotoxicity Analysis of Vaccine

*In vitro* cytotoxicity analysis results of DLDH and MSN-DLDH@HP55 are shown in **Figure 4B**. The toxicity of MSN in the control group was high. When the concentration of MSN was 20 µg/ml, the viability of YCK cells was only 71% (**Figure S3**), but the toxicity of the MSN-DLDH and MSN-DLDH@HP55 was decreased sharply. The viability of YCK cells was 80% after incubation with DLDH at the concentration of 180 µg/ml for 24 h, and viability of YCK cells was 67% while the DLDH concentration at 200 µg/ml. The viability of YCK cells was 79% after incubation with MSN-DLDH@HP55 at the concentration of 120 µg/ml for 24 h, and viability of YCK cells was lower than 70% while the DLDH concentration at 140 µg/ml or higher. The results showed that and MSN-DLDH@HP55 vaccine had good biocompatibility to YCK cells at concentration of 120 µg/ml.

### Relative Percent Survival Assays

**Figure 5:**
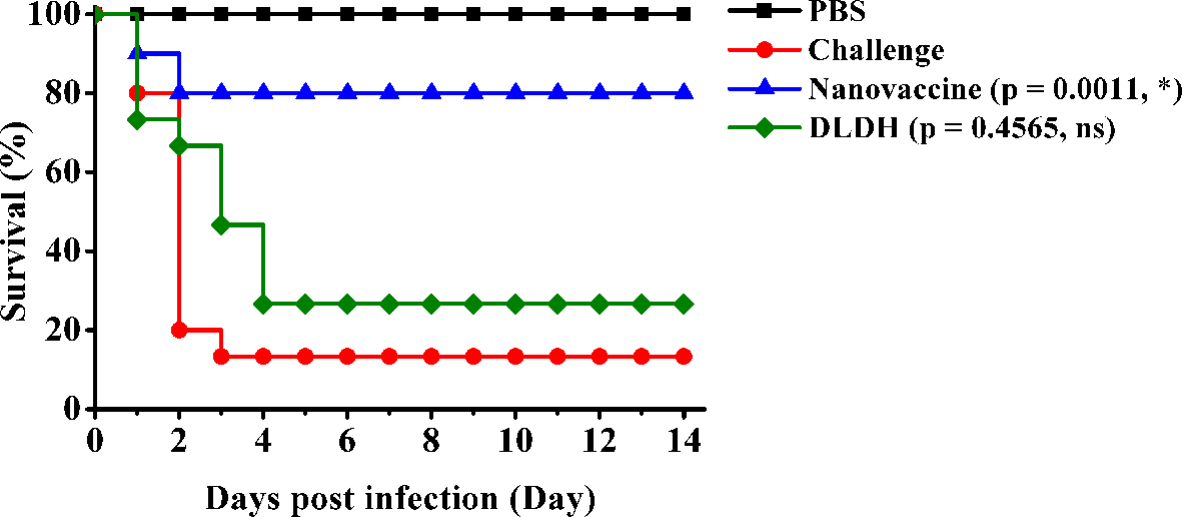
Relative percent survival analysis. The results showed survival curve of different experimental groups. Challenge, the unimmunized large yellow croakers were infected with *Vibrio alginolyticus* (1.1 × 10^7^ CFU/ml) as control. Nanovaccine group, the orally immunized fish were challenged with *Vibrio alginolyticus* (1.1 × 10^7^ CFU/ml). DLDH group, the negative control of oral vaccine was prepared by the same methods without MSNs carriers. PBS group, the immunized fish were injected with PBS buffer as control. The data of survival curve were analyzed by Gehan-Breslow-Wilcoxon test. *P < 0.05; ns, non-significant.

To assess the protection efficacy of the MSN-DLDH@HP55 vaccine post challenge, we performed the immune protection assay. The survival rate of the large yellow croaker in the challenge group 13.33% after inoculation with *Vibrio alginolyticus*, and the DLDH control group was 26.67%, while that in the nanovaccine group was 80% (**Figure 5**). Hence, the RPS of MSN-DLDH@HP55 vaccine and DLDH against large yellow croaker were 76.92% and 15.39%, suggesting that the MSNs delivery system could play a key role in delivering antigens. The results also demonstrated that the oral vaccine MSN-DLDH@HP55 prepared in this study had good immunoprotective effects against *Vibrio alginolyticus*.

### Analysis of Serum Antibody Levels

**Figure 6:**
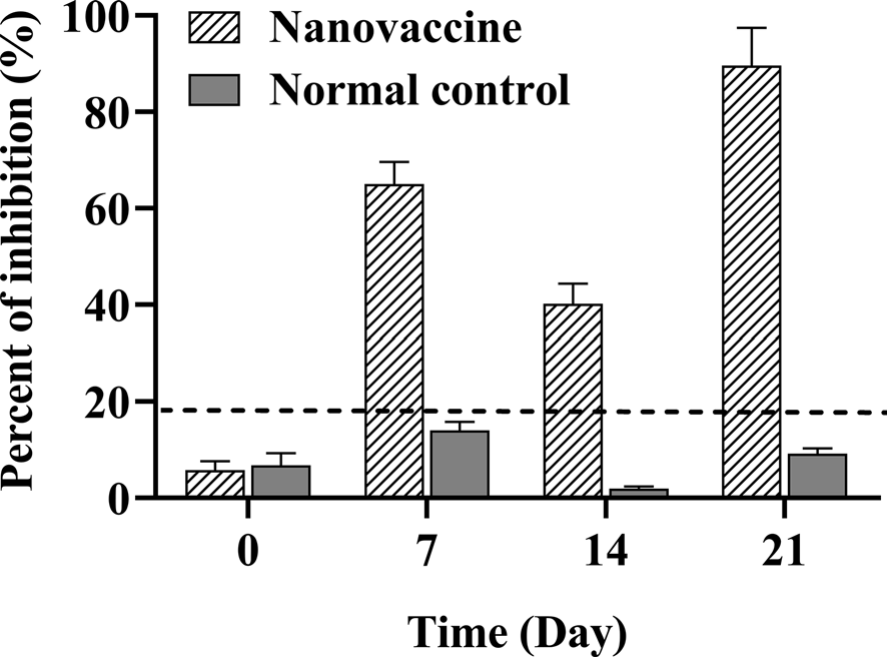
Serum antibody level of large yellow croaker was assessed at 7, 14, and 21 days after immunization with MSN-DLDH@HP55 vaccine. Blocking ELISA assay was used to detect the specific serum antibody against DLDH protein. When the absolute value of percent of inhibition (PI) was no less than 18.4% (dotted line), the sample was defined as positive. The preimmunized serum was as negative control (NC). The results of PI are presented as mean values (n=3) and error bars represent ± SEM.

The specific antibody response to vaccination increased gradually at days 7, 14, and 21 (**Figure 6**). The absolute value of antibody inhibition rate in the nanovaccine group preimmunized serum (NC) of large yellow croaker was lower than 6%. The absolute value of inhibition rate of antibody in nanovaccine group serum increased to 65.05% at 7 days, suggesting that the specific antibody against the DLDH protein was rising. The level of serum antibody in the nanovaccine group was slightly decreased at 14 days during the withdrawal period interval. After the secondary vaccination, the absolute value of inhibition rate of serum antibody in the nanovaccine group increased to 89.61% at 21 days, and OD_490_ value was 0.997. Our results showed that the MSN-DLDH carrier system could transfer DLDH antigen to the intestinal tract of large yellow croaker by oral administration, eliciting an immune response and producing specific antibodies against *Vibrio alginolyticus*.

### Analysis of the Expression Levels of Cytokines

**Figure 7:**
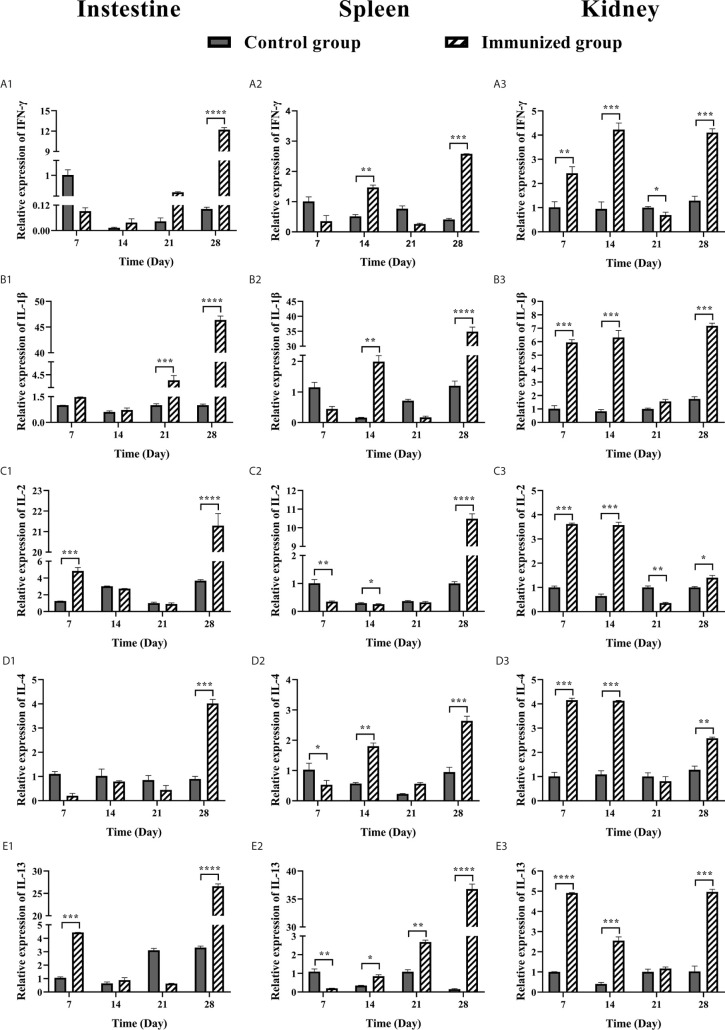
RT-qPCR analysis of cytokine expression (IFNγ, IL-1β, IL-2, IL-4, IL-13) in large yellow croaker after oral vaccination at 7, 14, 21, and 28 days. **(A)** The mRNA expression level of IFNγ in intestine, spleen, and head kidney tissues. **(B)** The mRNA expression level of IL-1β in intestine, spleen, and head kidney tissues. **(C)** The mRNA expression level of IL-2 in intestine, spleen, and head kidney tissues. **(D)** The mRNA expression level of IL-4 in intestine, spleen, and head kidney tissues. **(E)** The mRNA expression level of IL-13 in intestine, spleen, and head kidney tissues. The relative gene expression of each sample was compared with control using the 2^-ΔΔCt^ method. β-actin was used as an internal control. The data were analyzed by t test (GraphPad Prism 8, unpaired t test). The results of relative expression of samples are presented as mean values (n=3) and error bars represent ± SEM. *P < 0.05, **P < 0.01, ***P < 0.001, ****P < 0.001.

In order to better understand the immune mechanism of the immune system of large yellow croaker, we analyzed the expression level of IFNγ, IL-1β, IL-2, IL-4, and IL-13 cytokines in the intestine, spleen, and head kidney of large yellow croaker after immunization (**Figure 7**). The expression levels of IFNγ and IL-1β cytokines in the intestine of immunized group were significantly up-regulated 12-fold and 46-fold at 28 days after immunization respectively, while IL-1β was up-regulated at 21 days (P < 0.001). The expression levels of IL-2 and IL-13 in the intestine of immunized group were up-regulated at 7 and 28 days (P < 0.001, P < 0.0001; P < 0.001, P < 0.0001) and the 28 days have the highest expression levels with up-regulated 21-fold and 26-fold, respectively. The expression levels of IL-4 in the intestine of immunized group were up-regulated at 28 days (P < 0.001).

The IFNγ, IL-1β, IL-2, IL-4 IL-13 expression levels in spleen of large yellow croaker were shown in **Figures 7A2–E2**. The expression levels of IFNγ and IL-1β in the spleen of immunized group were up-regulated at 14 and 28 days (P < 0.01, P < 0.001; P < 0.01, P < 0.0001), meanwhile the IL-1β was up-regulated 35-fold at 28 days. The expression of IL-2 in the spleen of immunized group was up-regulated 10-fold at 28 days after immunization. The IL-4 was up-regulated at 14 and 28 days, down-regulated at 21 days (P < 0.01, P < 0.001), of which the highest expression level at 28 days. The IL-13 was up-regulated at 21 and 28 days after immunization (P < 0.01, P < 0.0001), and the 28 days was up-regulated 37-fold.

The expression levels of IFNγ and IL-1β in the head kidney of immunized group were up-regulated at 7, 14 and 28 days (P < 0.01, P < 0.001, P < 0.001; P < 0.001, P < 0.001, P < 0.001) and down-regulated at 21 days. The highest expression level of IFNγ was at 14 days, while the IL-1β was at 28 days. The expression of IL-2 in the head kidney of immunized group was up-regulated at 7 and 14 days after immunization (P < 0.001, P < 0.001), with the highest expression level at 7 days, while it was down-regulated at 21 and 28 days. The IL-4 and IL-13 in the head kidney of immunized group were up-regulated at 7 and 14 days (P < 0.001, P < 0.001; P < 0.0001, P < 0.001), down-regulated at 21 days, and up-regulated at 28 days (P < 0.01, P < 0.001). The highest expression level of IL-4 was at 7 days, and the IL-13 was at 28 days.

## Discussion

Vaccination has become one of the most important disease prevention and control strategies in modern aquaculture. Immunity *via* oral administration has proven to be widely applicable, efficient in time and cost, and greatly applicable to the safety management of fish of various sizes and stages (31–33). In this study, MSN was used to deliver antigen to prepare a high-load, pH controlled release, oral nanovaccine, which could reach the intestinal tract under the protection of enteric coatings, HP55, and directly pass through the intestinal epithelial cells to deliver antigen to Peyer patches, thereby interacting with immune cells to promote the induction of specific immune responses (34, 35). We analyzed the serum antibody levels of immunized large yellow croaker and the expression levels of IFNγ, IL-1β, IL-2, IL-4, and IL-13 in intestine, spleen, and head kidney. The results showed that the prepared oral vaccine could induce the innate and adaptive immune response of large yellow croaker.

Traditional oral vaccines are easily degraded in complex aquaculture and enzyme-rich gastrointestinal environments, and are not well transmitted to the hindgut or absorbed by immune cells, greatly affecting their immune protection effect. Surface fixation or adsorption of protein to the nanoparticles protects the antigens and thus improves the immune effect of oral vaccines (36). The whole three-dimensional structure of the DLDH protein like a funnel, and the maximum physical size was approximately 62 Å (6.2 nm) in length and 70 Å in width (7 nm), and the pore diameter of the nanoparticles was 7.83 nm, indicating that the antigen could be loaded into the pore channel. Previous studies have confirmed that the loading capacity of mesoporous materials is related to the specific surface area and pore size of the material. The larger the specific surface area and porosity, the stronger the loading capacity. In our study, the DLDH antigens could be loaded into MSN by physical adsorption and hydrogen bonding interactions (37, 38), and the protein loading capacity of the prepared MSN-DLDH was 42.16%, which is 728.91 μg of protein per mg of MSN. The higher protein loading capacity reduced the necessary quantity of MSN while maintaining a high concentration of antigen, thus reducing the toxicity and stimulating effects on fish cells. Although MSN was able to transport antigens to the intestine, it could not tolerate the negative effect of acidic stomach that could degrade antigens. Therefore, we used HP55 as an enteric coating to protect MSN-loaded antigen from the acidic environment and to control the release of antigen in weak alkaline environments (39). Thus, a large amount of antigens were delivered to the lamina propria of the hindgut, which provided an effective concentration for dendritic cells to uptake antigens (34, 40, 41). This prolonged the interaction between the antigen and the immune system, enhancing the immune response. Moreover, we also coated the surface of the vaccine-attached feed with chitosan to prevent the vaccine from releasing antigen in advance in the complex aquiculture environment. Chitosan is a non-toxic and degradable cationic polysaccharide with good absorption and adhesion. It can stimulate adaptive immune responses, including cellular and humoral immunity, and elicit effective immune response in oral mucosa and intestine of fish (42–44).

In recent years, nanoparticles were used as adjuvants and effective delivery systems for protective antigens, which not only enhanced the immunogenicity of weak antigens, but also facilitated the development of stable vaccine under complicated gastrointestinal environments. Different forms of nanoparticles have been widely studied for development of nanovaccines in aquaculture, including inorganic nanoparticles, polymeric nanoparticles, synthetically derived polymers, and lipid-based biomolecular nanoparticles (8, 12, 20). Mohamedi et al. (45) incorporated the antigen into the carrier system of ISCOMs and provided protective immunity to HSV-2 in mice *via* oral administration. The vaccine could promote systemic and local immune response in mice. However, ISCOMs were difficult to prepare, and the structure of the antigen must be modified to be loaded. Zhang et al. (17) developed a PMMMA-PLGA (PTRBL)/Trx-SIP oral vaccine against tilapia using biomedical material PLGA. Although the vaccine was of robust immune protective effect, the high cost of preparation made it difficult to apply clinically. The vaccine-loaded chitosan nanoparticles prepared by Sajal et al. (27) was lower cost than the PLGA vaccine, but it was necessary to add immersion immunity to increase the vaccine immune protection effect, which increased the difficulty of the immune process. In this study, the *E. coli* was used as a prokaryotic expression system to express antigen on a large scale, and the easy-to-prepare MSN material was used as a delivery system to prepare the MSN-DLDH@HP55 nanovaccine, which greatly reduced the cost of the vaccine. Furthermore, the protective antigen DLDH, had a highly conserved amino acid sequence (sequence homology up to 99%, data not shown) in *Vibrio*. It was the common protective antigen with cross-protection effect for the pathogen of *Vibrio alginolyticus*, *Vibrio parahaemolyticus*, and *Vibrio harveyi* (4). The antigenic epitopes were similar, thus providing the theoretical basis for the future development and industrialization of oral vaccines against *Vibrio* diseases. MSN nanoparticle delivery system prepared in this study was of wide applicability and could be used as a delivery system for various antigens (46, 47), paving the way for development of multiple oral vaccines for fish.

The immune system of fish is composed of innate and adaptive immunity (40). The innate immunity responds quickly after exposure to pathogens, producing a series of immune responses, including inflammatory reactions. The innate immune system of fish plays an important role in defending against pathogens. Moreover, innate immunity also plays an important role for acquired immunity as well as homeostasis (48). The intestine, spleen, and kidney are key tissues for fish immune response, and are rich in lymphoid cells (49). Epithelial cells in the intestinal mucosa directly deliver antigens to dendritic cells and initiate immune response during oral administration. Spleen and head kidney are capable of clearing soluble granular antigens and important analogues from blood (50). Enriched antigens may stimulate strong immune responses, resulting in the change of expression of immune-related immune factors in immune nodes. IFNγ is an important immunoregulatory molecule in innate and adaptive immunity processes. In the current study, the expression of IFNγ in the intestine, spleen, and head kidney of the immunized group was up-regulated at 14 or 28 days. This indicated that the MSN-DLDH@HP55 vaccine could induce the innate and adaptive response, promoting immune-related macrophage activation, Th1 response, and inflammatory response processes (51, 52). The IL-1β cytokine expression levels in the intestine, spleen, and head kidney of the immunized group were up-regulated at 28 days after immunization, suggesting activation of monocytes/macrophages, which proliferated and produced immune effectors (e.g., IL-1β), thereby exerting immunomodulatory effects (53, 54). The expression levels of the T cell growth factor IL-2 in the intestine, spleen, and head kidney of the immunized group were also up-regulated at 14 or 28 days after immunization. This implied that activated CD4^+^ T cells produced the IL-2 cytokine, promoted the differentiation of the CD4^+^ T cells into Th cells and other T cell subsets, and enhanced the immune response processes (55, 56). Controlling overactive inflammatory responses and preventing self-reactivity are important ways for the organism to limit its own damage (57). IL-4 and IL-13 are anti-inflammatory cytokines, which play key roles in preventing inflammation and autoimmune diseases (58). Our study showed that the expression levels of the IL-4 and IL-13 cytokines in the intestine, spleen, and head kidney of the immunized group were up-regulated at different times. Notably, this indicated that the vaccine could activate the adaptive immune system of large yellow croaker, induce the proliferation and activation of B cells, and stimulate the production of specific antibodies (31, 59, 60). Our study revealed that IFNγ, IL-1β, IL-4, and IL-13 cytokines in different tissues had the highest expression at different times, which may be related to the route of vaccination. Since we vaccinated fish for 7 consecutive days by oral feeding, the results suggested that innate immunity may be playing an important regulatory role in oral administration of fish, but the detailed mechanism needs further exploration. In addition, this study also confirmed changes in serum antibody levels in large yellow croakers at different time after vaccination. The results showed that oral immunization stimulated the steady increase of antibodies levels in large yellow croaker. The immune mechanism of fish is complicated. There are few studies focusing on the regulation mechanism of immune factors *via* oral vaccination. This study not only enriches the data of innate immune factors of fish nano-oral vaccines, but also confirms that the immunological load of nanoparticles with protective antigen can stimulate long-lasting innate and adaptive immunity. Interestingly, our MSN-DLDH@HP55 vaccine showed superior efficiency of protection. When the challenge dose was 1.1 × 10^7^ CFU/ml, the RPS of large yellow croaker against *Vibrio alginolyticus* was 76.92%, which have shown promising results. However, the nanovaccine should be further optimized in terms of the refinement of the vaccine formula ratio, the more suitable does of oral administration feeding, and the best immunization periods before clinical application.

The biosafety of nanoparticles has been a controversial issue in the field of nanotechnology, cytotoxicity of nanoparticles as carriers is critical while developing vaccines. The cytoxicity evaluation of nanovaccine particles cannot be determined by the toxicity of their original materials, which are absorbed by cells through different pathways, including pinocytosis and phagocytosis (61). Biocompatibility of MSN is not only related to material particle size, morphology, structure, dosage, surface properties, and quantity used, but are also closely related to immunized species, protein conformation, and surface charge (12, 21, 61). Mahony et al. (18) revealed that 0.1 mg/ml HMSN was toxic to MDBK cells, but 0.01 mg/ml was not. Duan et al. (62) also confirmed that silica nanoparticles were dose-dependent on toxicity to endothelial cell: 0.05 mg/ml silica nanoparticles significantly affected cell viability, and the minimum tolerable dose was 0.025 mg/ml. This study directly assessed the biosafety of a prepared nanovaccine delivery system in kidney cells of large yellow croaker. Interestingly, the results showed that nanoparticles had good biocompatibility to YCK cells at a higher concentration of 120 µg/ml, which may be closely related to the high antigen loading capacity. Although our study confirmed that relative higher doses (the clinical tests concentration of vaccine was of 40 µg/ml) of MSN-DLDH@HP55 were biocompatible to YCK cells, the use of MSN nanoparticles in fish vaccines is still a relatively new area, and additional clinical tests are required for vaccine biosafety evaluation. Systematic exploration of the effects of various factors on the MSN nanomaterials and evaluation of its long-term *in vivo* mechanism will be the focus of future work.

In summary, the nanovaccine delivery system prepared in this study has the advantages of high loading capacity, pH controlled release of antigen, good biocompatibility, and cost efficiency. It can stimulate long-lasting innate and adaptive immunity, implying that MSN is a potential vaccine delivery system for fish. This study, thus, provides a theoretical foundation for industrialization of oral vaccine against *Vibrio* species for large yellow croaker using this MSN delivery system. It also provides new ideals and directions for development of vaccines with good stability and biocompatibility under a complicated gastrointestinal environment.

## Data Availability Statement

The datasets presented in this study can be found in online repositories. The names of the repository/repositories and accession number(s) can be found in the article/**Supplementary Material**.

## Ethics Statement

The animal study was reviewed and approved by The Research Ethics Committee of Institute of Biotechnology, Fujian Academy of Agricultural Sciences.

## Author Contributions

YW: conceptualization, funding acquisition, project administration, writing—review and editing. WZ: methodology, software, validation, formal analysis, investigation, writing—original draft. CZ: conceptualization, methodology, project administration, formal analysis, writing—review and editing. FX and FC: methodology. XL: formal analysis, writing—review and editing. HG: methodology, writing—review and editing, funding acquisition, resources. All authors contributed to the article and approved the submitted version.

## Funding

This work was supported by the National Thousand Talents Program of China, the STS Project of Chinese Academy of Sciences and Fujian Province (2016T3041), the Key Project of Fujian Province (2017N0031), the Fujian Province Natural Science Foundation of China (2019J01280), Special Funds of the Central Government Guiding Local Science and Technology Development (2020L3008), National Key R&D Program of China (2019YFD0900102), Fujian Provincial Public Research Institute of Fundamental Research (2019R1026-4).

## Conflict of Interest

The authors declare that the research was conducted in the absence of any commercial or financial relationships that could be construed as a potential conflict of interest.

## Supplementary Material

Supplementary Figure 1 The three-dimensional structure of DLDH. The whole three-dimensional structure of the DLDH protein like a funnel, and the maximum physical size was approximately 62 Å in length and 70 Å in width.

Supplementary Figure 2 Dynamic light scattering analysis of MSN and MSN-DLDH. Particle size analysis of **(A)** MSN and **(B)** MSN-DLDH. Potential analysis of **(C)** MSN and **(D)** MSN-DLDH. The samples were dissolved in ddH_2_O and measured at 25°C.

Supplementary Figure 3 Analysis of cytotoxicity of MSN *in vitro* by MTT assay. Cell viability of YCK cells was assessed after incubation with different concentrations (0, 0.4, 0.8, 2, 4, 6, 8, 10, and 20 µg/ml) of MSN for 24 h, respectively. The results of cell viability are presented as mean values (n=3) and error bars represent ± SEM.

Supplementary Table 1 Elemental analysis of MSN and MSN-DLDH.
