# Supplementary material for: pH-Controlled Release of Antigens Using Mesoporous Silica Nanoparticles Delivery System for Developing a Fish Oral Vaccine
Source: Front Immunol. 2021 Apr 19;12:644396. doi: 10.3389/fimmu.2021.644396 (PMC8089398; doi:10.3389/fimmu.2021.644396)
Supplement: Supplementary Table 1 — Elemental analysis of MSN and MSN-DLDH. [file Table_1.doc]

**Table S1. Elemental analysis of MSN and MSN-DLDH.**

| Sample | C% | H% |
| --- | --- | --- |
| MSN | 0.95 | 0.96 |
| MSN-DLDH | 11.12 | 2.85 |
